# Sero-prevalence of Hepatitis B and C viral co-infections among HIV-1 infected ART-naïve individuals in Kumasi, Ghana

**DOI:** 10.1371/journal.pone.0215377

**Published:** 2019-04-19

**Authors:** Richard Boateng, Mohamed Mutocheluh, Albert Dompreh, Dorcas Obiri-Yeboah, Enoch Odame Anto, Michael Owusu, Patrick Williams Narkwa

**Affiliations:** 1 Department of Clinical Microbiology, School of Medical Sciences, Kwame Nkrumah University of Science and Technology, Kumasi, Ghana; 2 Department of Clinical Microbiology, Komfo Anokye Teaching Hospital, Kumasi, Ghana; 3 Department of Microbiology and Immunology, School of Medical Sciences, University of Cape Coast, Cape Coast, Ghana; 4 School of Medical and Health Sciences (SMHS), Edith Cowan University (ECU), Perth, Western Australia, Australia; 5 Department of Medical Laboratory Technology, Faculty of Allied Health Sciences, Kwame Nkrumah University of Science and Technology, Kumasi, Ghana; University of Cincinnati College of Medicine, UNITED STATES

## Abstract

**Background:**

The study assessed the hepatitis B virus (HBV) and hepatitis C virus (HCV) co-infection paradigm among the human immunodeficiency virus (HIV) infected patients attending a tertiary hospital in Ghana. Also, the immunological and virological characterisation of these viruses, prior to antiretroviral therapy (ART) initiation was investigated.

**Method:**

A total of 400 HIV infected (HIV type-1) treatment naïve subjects ≥18 years were enrolled and tested for HBsAg and anti-HCV. Hepatitis B virus serological profile was performed on samples that were HBV positive. CD4+ T-cell count and HIV-1 RNA viral loads were determined using BD FacsCalibur analyzer (USA) and COBAS AmpliPrep/COBAS TaqMan Analyzer (USA) respectively.

**Results:**

The overall prevalence of HBV/HCV co-infection among the HIV-1 patients was 18.0%. The prevalence of HIV-HBV and HIV-HCV co-infections were 12.5% and 5.5% respectively. The prevalence of active viral hepatitis (HBeAg-positive) among HIV-HBV co-infected patients was 40%. None of the patients had anti-HBc IgM. HIV-HBV co-infection was associated with lower CD4+ T-cell count as well as higher HIV-1 viral load compared to both HIV mono- infection and HIV-HCV co- infection (*p*<0.05) respectively. HBeAg positivity was associated with severe immunosuppression and higher HIV viral load. Patients aged 18–33 years [aOR = 9.66(1.17–79.61); *p* = 0.035], male gender [aOR = 2.74(1.15–6.51); *p* = 0.023], primary education [aOR = 9.60(1.21–76.08); *p* = 0.032], secondary education [aOR = 14.67(1.82–118.08); *p* = 0.012] and being single [aOR = 2.88(1.12–7.39); *p* = 0.028] were independent risk factors of HIV-HBV co-infections but not HIV-HCV co-infections.

**Conclusion:**

The present study highlights the predominance of HBV exposure among the HIV infected patients in Ghana. HBV coinfection was associated with severe immunosuppression and higher HIV-1 viral load.

## Introduction

The current study was motivated by reports that data on individuals co-infected with HIV and viral hepatitis in West Africa was still emerging [1–3]. Moreover, there has been conflicting reports on the effects of viral hepatitis on the immunity of HIV patients co-infected with either HBV or HCV. Whereas some studies showed HBV and HCV coinfections were linked to a more severe form of immunosuppression of pre ART CD4+ T-cell counts compared to those with HIV mono-infection [4–6] others observed no differences [7, 8].

Globally, about 400 million people are infected with hepatitis B virus and 180 million are infected with hepatitis C virus. Both infections account for 60% of cirrhosis and 80% of hepatocellular carcinoma and also cause one million deaths worldwide each year, mostly in poor countries [9].

Reports suggested that both viral hepatitis infections are associated with more rapid progression of liver fibrosis and fibrogenesis during HIV co-infection and liver pathology has become the leading causes of death in some countries [10–12]. Moreover, it has been estimated that about 30% of people with HIV are coinfected with HCV or HBV worldwide [13].

The global prevalence rate of HIV/HBV and HIV/ HCV co-infections in sub-Saharan African countries were reported as 15% and 7%, respectively [14]. In Ghana, the rate was at 13% and 3.6% respectively [15]. However, previous studies have reported that the prevalence rates of HBV and HCV in HIV negative Ghanaian population were reported as 8–15% [16] and 3–5% [17] respectively; indicating a clear viral hepatitis endemicity in Ghana.

The advent of ART has had a major impact on HIV-associated mortality in resource constrained countries resulting in HIV becoming a chronic condition. Co-morbidities like HBV and HCV infections currently pose major clinical and public health challenges [18, 19]. For example; the management and monitoring of HCV is not yet integrated in public ART programs in Ghana and many sub-Saharan countries, although such information is essential to position the need to prioritise the provision of treatment and to develop evidence-based guidelines and policies.

Importantly, HBV and HCV infections have been correlated with several clinical manifestations in HIV-infected patients including impaired immune response during ART and increased susceptibility to ART-related liver toxicity [12]. These interactions uphold the importance of timely screening of one infection in the presence of the other. Hence, prior to the implementation of the “treat all” policy which requires that all HIV positive clients be offered ART, HBV/HIV coinfection was among the eligibility criteria. Such co-infected clients were being prioritized for ART irrespective of CD4 count. Co-infection with HBV also affected the ART regimen and is part of the justification for the preferred ART regimen in Ghana being an Efavirenz and Tenofovir based regimen. The regimen of Tenofovir plus lamivudine plus Efavirenz ensures that such co-infected client avoid Nevirapine due to its potential hepatotoxicity while also receiving 2 drugs which are used in the treatment of HBV infection. Therefore, the current study determined the prevalence of HBV and HCV in patients naïve to ART and infected with HIV in the middle belt of Ghana and also assessed the effects of viral hepatitis on the immunity of HIV patients co-infected with either HBV or HCV.

## Method

### Sample collection and processing

This analytical cross-sectional study was conducted at the HIV Clinic in Ghana’s second largest tertiary hospital, called the Komfo Anokye Teaching Hospital (KATH) in Kumasi, Ghana from May 2016 to April 2017. KATH is a tertiary referral hospital with a 1200 bed capacity and serves as the main Teaching Hospital of the School of Medical Sciences of the Kwame Nkrumah University of Science and Technology (KNUST). KATH serves patients from a wider geographical area including referrals from northern, western and eastern parts of Ghana. The HIV treatment centre is run under the Directorate of Internal Medicine, started in the year 2003, offers comprehensive care for HIV patients and has registered over 10,000 HIV-infected patients on OPD basis. A simple randomise sampling technique was used to recruit a total of 400 HIV-1 treatment naïve subjects ≥18 years. The volunteers were recruited after providing written consent. Recruitment of subjects was performed during their routine clinic visit days. During such days, previous clinical history of subjects was assessed and patients that fell within the criteria for this study identified. The study inclusion criteria were: age ≥ 18 years, patients with HIV-1 infection and patients who agreed to take part in the study. The study exclusion criteria were: patients with HIV-2 or HIV1/2 co-infection and patients not willing or unable to take part in the study.

Once a subject qualified for the study, the rationale and objectives as well as risk factors involved were explained to the subject. Only participants who agreed to the study were included. Elaborate pilot-tested questionnaire designed by reviewing previous studies of similar objective and tailored to fit our study objectives was used to obtain subject’s socio-demographical data. Venous blood sample (5ml) was collected aseptically into K_3_EDTA tube for T lymphocyte cell count, viral load (HIV-1 RNA) and viral serology tests. CD4+ T cell count and HIV-1 RNA viral loads were determined using BD FacsCalibur analyzer (BD Biosciences, USA) and COBAS AmpliPrep/COBAS TaqMan Analyzer (Roche Diagnostics, USA) respectively. Hepatitis status (HBsAg) and anti-HCV of subjects were determined using One Step test trip (InTec PRODUCTS, INC., Fujian P. R. China). Hepatitis B profile was performed using Hepatitis B profile kit (DiaSpot Rapid Diagnostic, Indonesia).

### Ethical considerations

Ethical approval for this study was obtained from the Committee on Human Research Publication and Ethics (CHRPE) of the School of Medical Sciences, Kwame Nkrumah University of Science and Technology (CHRPE/AP/483/17) and also from the Research and Development Department of KATH. Written informed consent was obtained from all participants after the aim and objectives of the study had been explained to them.

### Data analysis

All categorical data were presented as frequency (percentages) and Chi square and Fishers exact test statistic were used to test for association were applicable. One-way ANOVA and Kruskal-Wallis one-way ANOVA were used to test for significance of differences among parametric and non-parametric variables respectively, followed by appropriate *posteriori* tests. Mann-Whitney U test and Independent sample t-test were used to compare non-parametric and parametric variables respectively among HIV-HBV co-infected population with respect to HBeAg status.

Multivariate logistic regression analysis was done to determine the odds of socio-demographics factors in predicting HIV-HBV and HIV-HCV co-infections. A p value < 0.05 was considered statistically significant. All statistical analyses were performed using IBM SPSS 25.0 Statistics and GraphPad Prism 7 version 7.04.

## Results

A total of 400 HIV-1 treatment naïve subjects successfully participated in the study comprising of 162 (40.5%) males and 238 (59.5%) females. The overall mean age of both sexes was 40.94 years (18–64) years. Majority (49.5%) of the subjects were married, attained at least primary level of education (71.5%), were employed (73.5%) and (68.0%) were living in the urban areas. The overall prevalence of viral hepatitis (HBV/HCV) was 18.0% (72/400). HIV-HBV co-infection was 12.5% (50/400) of which 28/50 (56%) were females and 22/50 (44.0%) were males whereas that of HIV-HCV co-infection was 5.5% (22/400) of which 16/22 (72.72%) were females and 6/22 (27.27%) were males. HIV mono-infection prevalence was 82.0% (328/400). Prevalence rate of both HIV-HCV and HIV-HBV concurrent infection was high among females than males. None had trio infection. Regarding the risk factors for HIV-HBV or HIV-HCV co-infection, there was significant association between the age, gender, level of education and marital status variables and HIV-HBV co-infection, however, the predictive capacity of socio-demographic variables for HIV-HCV co-infections were insignificant. Participants who were 18–33 years (aOR = 9.66, 95%CI (1.17–79.61), *p*-value = 0.035), male gender (aOR = 2.74, 95%CI (1.15–6.52), *p*-value = 0.023), had primary school education (aOR = 9.60, 95%CI (1.21–76.08), *p*-value = 0.032), secondary school education (aOR = 14.67, 95%CI (1.82–118.09), *p*-value = 0.012) and participants who were single (aOR = 2.88, 95%CI (1.12–7.39), *p*-value = 0.028) all had higher odds of having HIV-HBV co-infections (Table 1).

**Table 1:** Socio-demographic characteristics of study subjects and risk factors for HIV-HBV or HIV-HCV Co-infection.

Characteristics	Total (N = 400)	HIV-1 mono (N = 328)	HIV-1/HBV (N = 50) ‡	aOR (95% Cl)	HIV-1/HCV (N = 22) †	aOR (95% Cl)
Age (years)						
18–33	100(25)	84(25.6)	18(36)‡‡	9.66(1.17–79.61)*	4(18.20)	0.58(0.09–3.66)
34–49	210(52.5)	165(50.3)	30(60)	7.33(0.94–57.31)	12(54.54)	0.85(0.20–3.55)
50–64	90(22.5)	79(24.1)	2(4)	1	6(27.27)	1
Gender						
Male	162(40.5)	134(40.8)	22(44)	2.74(1.15–6.52)*	6(27.27)	1.13(0.29–4.45)
Female	238(59.5)	194(59.1)	28(56)	1	16(72.72)	1
Level of Education						
Primary	164(41)	132(40.2)	24(48)‡‡‡	9.60(1.21–76.08)**	8(36.36)	0.68(0.16–2.84)
JHS	8(2)	2(0.6)	6(12)	15.6 (9.62–18.61)***	0(0)	-
SHS	98(24.5)	76(23.2)	16(32)	14.67(1.82–118.09)*	6(27.27)	0.79(0.17–3.73)
Tertiary	16(4)	14(4.3)	2(4)	8.00(0.45–142.66)	0(0)	-
No formal Education	114(28.5)	104(31.7)	2(4)	1	8(36.36)	1
Marital status						
Married	198(49.5)	168(51.2)	20(40)‡‡	1	10(45.45)	1
Single	90(22.5)	62(18.9)	22(44)	2.88(1.12–7.39*	6(27.27)	1.34(0.31–5.88)
Widow/Divorced	112(28)	98(29.9)	8(16)	0.69(0.20–2.29)	6(27.27)	1.06(0.25–4.63)
Residence						
Urban	272(68)	224(68.3)	36(72)	1	12(54.54)	1
Peri-Urban	128(32)	104(31.7)	14(28)	0.84(0.43–1.62)	10(45.45)	1.79(0.75–4.29)
Employment						
Employed	294(73.5)	248(75.6)	34(68)	1	12(54.54)	1
Unemployed	106(26)	80(24.4)	16(32)	1.46(0.77–2.78)	10(45.54)	2.58(1.08–6.20)

Chi square and Fisher exact test (2x2 contingencies) was performed to compare categorical variables. p < 0.05 was considered statistically significant.

‡ for comparison between HIV-1 mono-infection and HIV-1/HBV co-infection

‡‡, p<0.01

‡‡‡, p<0.0001.

† for comparison between HIV-1 mono-infection and HIV-1/HCV co-infection

aOR: adjusted odds ratio; Covariate for adjusted model in age, level of education and marital status. p < 0.05 was considered statistically significant (p values of significant variables are in bold print). 1: reference category

*, p<0.05

**, p<0.01

***, p<0.0001

The overall median CD4+ T T-cell count was 343.0 cells/μL (range, 198.5–572.3 cells/μl) as summarised in Table 2. Study participants with HIV-HBV co-infection had statistically significant lower CD4+ T-cell count (213.0 cells/μl) compared to HIV mono-infected participants (386.0 cells/μl) (*p*<0.0001) and also compared to HIV-HCV co-infected patients (490.0 cells/μl) (*p* = 0.013).The mean HIV-1 viral load was 4.87log_10_ copies/mL and was significantly higher among participants with HIV-HBV co-infection compared to HIV-HCV co-infection (5.39 vs 4.50 log_10_ copies/mL respectively) (*p* = 0.011) as well as participants with HIV mono-infection (5.39 vs 4.82 log_10_ copies/mL respectively) (*p*<0.0001) (Table 2).

**Table 2:** Immunological and virological parameters of the study population.

Parameter	CD4+ T lymphocyte (cells/μl)	Log [HIV Viral Load] (copies/mL)
Group	Median (IQR)	Mean ± SD
Overall	343.0 (198.5–572.3)	4.87 ± 0.81
HIV-HBV	213.0 (127.5–360.0)*	5.39 ± 0.94*
HIV-HCV	490.0 (213.0–543.0)*	4.50 ± 0.72*
HIV mono	386.0 (211.5–615.8)*	4.82 ± 0.76*
*p-*value	**<0.0001**	**<0.0001**
Significant pair	HIV-HBV vs HIV-HCV and HIV-HBV vs HIV mono	HIV-HBV vs HIV-HCV and HIV-HBV vs HIV mono

* indicates significant difference compared to each other. IQR: interquartile range; SD: standard deviation; Kruskal-Wallis one-way ANOVA for differences between non-parametric variables (CD4+ T lymphocyte) followed by Bonferroni post hoc test; One-way ANOVA for differences between parametric variables (Log [HIV Viral Load]) followed by Turkey Honestly Significant Difference. p < 0.05 was considered statistically significant.

Taken together, the HIV-HBV participants had the lowest CD4+ cells count and also the highest viral load compared to the HIV-HCV and the HIV mono-infected participants.

**Table 3** shows immunological and virological parameters of HIV-HBV infected patients. HIV-HBV co-infected patients with positive HBeAg had statistically significant lower CD4+ T-cell count (218.0 cells/μl vs 349.5 cells/μl; *p*-value = 0.031) and higher viral load (5.67 copies/mL vs 4.83 copies/mL; *p-*value = 0.007) compared to the negative HBeAg counterparts.

**Table 3:** Immunological and virological parameters of HIV-HBV infected participants.

	HBeAg status				
Results	Positive		Negative		
Parameter	Mean	Median(IQR)	Mean	Median(IQR)	*p*-value
CD4+ T lymphocyte count	228.5	218.0(103.8–344.0)	415.19	349.5(143.0–384.0)	**0.031**†
Log (HIV Viral Load)	5.67	5.73(4.9–6.7)	4.83	4.88(4.3–5.9)	**0.007‡**

†Mann-Whitney U test was used to compare non-parametric variables

‡Independent sample t-test for parametric variables (log [HIV viral load]).

p < 0.05 was considered statistically significant

Majority, (44.0%) of the patients with HIV-HBV co-infection were categorized as WHO clinical stage 4. In contrast, 36.4% and 31% of the patients with HIV-HCV co-infection and HIV mono-infection respectively were categorized as WHO clinical stage 1.

## Discussion

Individuals with HIV infection could live longer in the era of increased access to ART but that could be complicated because many are coinfected with viral hepatitis B and or C in sub-Saharan Africa. Viral hepatitis B and or C coinfected HIV-patients have been associated with increased immunosuppression pre-ART during ART and also increased ART-related liver toxicity [12] although some studies reported to the contrary [8]. Therefore, the current study sought to establish the burden of viral hepatitis B and C among ART naïve HIV patients and to herald the deleterious effects of these hepatitis viruses.

The current study showed the prevalence of HIV-HBV and HIV-HCV co-infections rate at 12.5% and 5.5% respectively. In line with our results a recent meta-analysis survey on HBV and HIV coinfection showed the rate at between 0% to as high as 28% in sub-Saharan Africa with higher rates being reported in West African countries (median: 11.5%); East African countries were found to have the lowest rates (median: 4.1%) [20]. More so, Sagoe *et al*., found HBV and HCV co-infection among HIV-infected persons in Ghana at 13.0% and 3.6% respectively consistent with findings from the current study and from Hamza *et al*., and Muriuki *et al*., in Nigeria and Kenya respectively [21, 22]. To the contrary, the seroprevalence of HIV/HBV and HIV/HCV coinfections was reported lower in Ethiopia, than in the rest of sub-Saharan Africa [23].

Taken together, results from the current study was either consistent or in line with many reported studies in sub-Saharan Africa.

HBeAg is a protein from the hepatitis B virus that circulates in infected blood when the virus is actively replicating. The presence of HBeAg suggests that the person is infectious and is able to spread the virus to other people.

In line with our expectation, the current study showed HBeAg was associated with severe immunosuppression and increased viral load (Table 3) consistent with a previous study in Ghana [3]. But that previous Ghanaian study showed a higher HBeAg of 55.6% contrary to the 44.4% shown in the current study. However, in contrast with the current study, Manyazewal *et al*., reported a very low rate of 0.8% (HBeAg) among HIV infected individuals in Ethiopia [23].

Consistent with many studies within Africa none of our study patients tested positive for anti-HBc IgM [15, 23, 24].

The observed diverse HBeAg status across different countries were associated with the diversity of patients from different population groups and geographical regions and sample sizes. Taken together, these reports suggest a significant number of HIV/HVB co-infected patients have the additional burden of acute HBV infection giving a compelling reason for policy makers in resource limited countries to include viral hepatitis B and C in their national ART programmes.

Consistent with reports from around the world [15, 22–25], the current study showed patients in the HIV/HBV groups were severely immunosuppressed and had higher HIV load (Tables 2 and 3) compared to their counterpart HIV/HCV and HIV monoinfection groups respectively. More so, several other studies have suggested that CD4+ T-cell count <200 cells/mm^3^ is often associated with higher HBV DNA levels [25–27] and increased risk for liver-related mortality [28].

Contrary to our expectations immunity was boosted as indicated by increased CD4+ T-cell count in the HIV/HCV group compared to that of the HIV monoinfection group and even the rate was twice as high compared to the HIV/HBV group; although a similar viral load was recorded in the HIV/HCV and HIV monoinfection respectively (Table 2). One school of thought could be that HCV inhibited or reduced CD4+ T-cells clearance by HIV. Another school of thought could be that HCV stimulated the activation of CD4+ T-cells as reported previously [29].

Majority of the HBV coinfected patients had advanced HIV disease (WHO stage 3 or 4) compared to their HIV monoinfected counterparts giving credence to the suggestion that HBV mediated immunosuppression and consistent with a report from Tanzania [12].

Socio-demographic characteristics such as age (18–33 years), male gender, primary and secondary levels of education as well as marital status (being single) posed a greater risk of acquiring HIV-HBV and HIV-HCV co-infection in this study (Table 1). HBV infection is mostly transmitted by means of sexual routes or perinatally [30]. The fact that male patients are more prone towards acquiring such coinfection could mainly be attributable to their higher rate of sexual promiscuity and their predominance as HBV carriers [16]. Risk factors for HIV-HCV co-infections could not be determined due to extremely low numbers.

The limitation of the current study was our inability to confirm HBV and HCV with molecular techniques and perform genotyping studies. The stated limitation was mainly due to financial constrains as the work was funded by the authors themselves.

## Conclusion

The present study highlights the predominance of HBV exposure among the HIV infected patients in Ghana. HBV coinfection was associated with severe immunosuppression and higher HIV-1 viral load compared with the HIV/HCV and HIV monoinfection groups respectively. Majority of the HIV/HBV group had chronic HBV infection. HCV surprisingly boosted immunity but not HIV viral load. Socio-demographic factors such as age (18–33 years), male gender, having primary and secondary education and being single was associated with increased risk of HIV-HBV co-infection.

Also, results from the current study could lead to policy change that would improve effective management of these patients and put to rest the conflicting reports of the deleterious effects or otherwise of HBV on the immunity of ART-naïve HIV coinfected patients.

## References

[1] Adewole OO, Anteyi E, Ajuwon Z, Wada I, Elegba F, Ahmed P, et al Hepatitis B and C virus co-infection in Nigerian patients with HIV infection. The journal of infection in developing countries. 2009;3(05):369–75. 19759507 10.3855/jidc.245

[2] Laurent C, Bourgeois A, Mpoudi‐Ngolé E, Kouanfack C, Ciaffi L, Nkoue N, et al High rates of active hepatitis B and C co‐infections in HIV‐1 infected Cameroonian adults initiating antiretroviral therapy. HIV medicine. 2010;11(1):85–9. 10.1111/j.1468-1293.2009.00742.x 19659944

[3] Noubiap JJN, Aka PV, Nanfack AJ, Agyingi LA, Ngai JN, Nyambi PN. Hepatitis B and C co-infections in some HIV-positive populations in Cameroon, West Central Africa: analysis of samples collected over more than a decade. PloS one. 2015;10(9):e0137375 10.1371/journal.pone.0137375 26371878 PMC4570762

[4] Diop‐Ndiaye H, Touré‐Kane C, Etard J-F, Lo G, Diaw P, Ngom‐Gueye N, et al Hepatitis B, C seroprevalence and delta viruses in HIV‐1 Senegalese patients at HAART initiation (retrospective study). Journal of medical virology. 2008;80(8):1332–6. 10.1002/jmv.21236 18551596

[5] Otegbayo JA, Taiwo BO, Akingbola TS, Odaibo GN, Adedapo KS, Penugonda S, et al Prevalence of hepatitis B and C seropositivity in a Nigerian cohort of HIV-infected patients. Annals of hepatology. 2008;7(2):152–6. 18626434

[6] van Griensven J, Phirum L, Choun K, Thai S, De Weggheleire A, Lynen L. Hepatitis B and C co-infection among HIV-infected adults while on antiretroviral treatment: long-term survival, CD4 cell count recovery and antiretroviral toxicity in Cambodia. PloS one. 2014;9(2):e88552 10.1371/journal.pone.0088552 24533106 PMC3922870

[7] Harania RS, Karuru J, Nelson M, Stebbing J. HIV, hepatitis B and hepatitis C coinfection in Kenya. Aids. 2008;22(10):1221–2. 10.1097/QAD.0b013e32830162a8 18525268

[8] da Silva CM, de Peder LD, Guelere AM, Horvath JD, Silva ES, Teixeira JJV, et al Seroprevalence of hepatitis B virus (HBV) and hepatitis C virus (HCV) among human immunodeficiency virus (HIV)-infected patients in an HBV endemic area in Brazil. PloS one. 2018;13(9):e0203272 10.1371/journal.pone.0203272 30192795 PMC6128547

[9] WHO. Global policy report on the prevention and control of viral hepatitis in WHO Member States 2013 [Available from: http://appswhoint/iris/bitstream/10665/85397/1/9789241564632_engpdf.

[10] Hernandez MD, Sherman KE. HIV/HCV coinfection natural history and disease progression, a review of the most recent literature. Current opinion in HIV and AIDS. 2011;6(6):478 10.1097/COH.0b013e32834bd365 22001892 PMC3293393

[11] Weber R, Sabin CA, Friis-Moller N, Reiss P, El-Sadr WM, Kirk O, et al Liver-related deaths in persons infected with the human immunodeficiency virus: the D: A: D study. Arch Intern Med. 2006;166(15):1632–41. 10.1001/archinte.166.15.1632 16908797

[12] Kilonzo SB, Gunda DW, Kashasha F, Mpondo BC. Liver fibrosis and Hepatitis B coinfection among ART Naive HIV-infected patients at a tertiary level hospital in Northwestern Tanzania: A cross-sectional study. Journal of tropical medicine. 2017;2017. 10.1155/2017/5629130 PMC5554579 28828009

[13] Lacombe K, Rockstroh J. HIV and viral hepatitis coinfections: advances and challenges. Gut. 2012;61:47–58. 10.1136/gutjnl-2012-302062 22504919

[14] Barth RE, Huijgen Q, Taljaard J, Hoepelman AI. Hepatitis B/C and HIV in sub-Saharan Africa: an association between highly prevalent infectious diseases. A systematic review and meta-analysis. International Journal of Infectious Diseases. 2010;14(12):e1024–e31. 10.1016/j.ijid.2010.06.013 20870439

[15] Sagoe KWC, Agyei AA, Ziga F, Lartey M, Adiku TK, Seshi M, et al Prevalence and impact of hepatitis B and C virus co‐infections in antiretroviral treatment naïve patients with HIV infection at a major treatment center in Ghana. Journal of medical virology. 2012;84(1):6–10. 10.1002/jmv.22262 22095533

[16] Mutocheluh M, Owusu M, Kwofie TB, Akadigo T, Appau E, Narkwa PW. Risk factors associated with hepatitis B exposure and the reliability of five rapid kits commonly used for screening blood donors in Ghana. BMC research notes. 2014;7(1):873. 25475050 10.1186/1756-0500-7-873 PMC4295511

[17] Agyeman AA, Ofori-Asenso R, Mprah A, Ashiagbor G. Epidemiology of hepatitis C virus in Ghana: a systematic review and meta-analysis. BMC infectious diseases. 2016;16(1):391. 27507267 10.1186/s12879-016-1708-7 PMC4977883

[18] Kourtis AP, Bulterys M, Hu DJ, Jamieson DJ. HIV–HBV coinfection—A global challenge. New England Journal of Medicine. 2012;366(19):1749–52. 10.1056/NEJMp1201796 22571198 PMC4453872

[19] Ocama P, Seremba E. Management of HIV and hepatitis C virus infections in resource-limited settings. Current opinion in HIV and AIDS. 2011;6(6):539–45. 10.1097/COH.0b013e32834bd23f 21918435

[20] Stabinski L, O'connor S, Barnhart M, Kahn RJ, Hamm TE. Prevalence of HIV and hepatitis B virus co-infection in sub-Saharan Africa and the potential impact and program feasibility of hepatitis B surface antigen screening in resource-limited settings. JAIDS Journal of Acquired Immune Deficiency Syndromes. 2015;68:S274–S85. 10.1097/QAI.0000000000000496 25768867 PMC10426262

[21] Hamza M, Samaila AA, Yakasai AM, Babashani M, Borodo MM, Habib AG. Prevalence of hepatitis B and C virus infections among HIV-infected patients in a tertiary hospital in North-Western Nigeria. Nigerian Journal of Basic and Clinical Sciences. 2013;10(2):76.

[22] Muriuki BM, Gicheru MM, Wachira D, Nyamache AK, Khamadi SA. Prevalence of hepatitis B and C viral co-infections among HIV-1 infected individuals in Nairobi, Kenya. BMC research notes. 2013;6(1):363. 24016453 10.1186/1756-0500-6-363 PMC3844558

[23] Manyazewal T, Sisay Z, Biadgilign S, Abegaz WE. Hepatitis B and hepatitis C virus infections among antiretroviral-naive and-experienced HIV co-infected adults. Journal of medical microbiology. 2014;63(5):742–7. 24757219 10.1099/jmm.0.063321-0

[24] Puglia M, Stasi C, Da Frè M, Voller F. Prevalence and characteristics of HIV/HBV and HIV/HCV coinfections in Tuscany. Brazilian Journal of Infectious Diseases. 2016;20(4):330–4. 10.1016/j.bjid.2015.11.007 26748234 PMC9427616

[25] Saha D, Pal A, Biswas A, Panigrahi R, Sarkar N, Sarkar J, et al Characterization of treatment-naive HIV/HBV co-infected patients attending ART clinic of a tertiary healthcare centre in eastern India. PLoS One. 2013;8(8):e73613 10.1371/journal.pone.0073613 24023688 PMC3758335

[26] Jobarteh M, Malfroy M, Peterson I, Jeng A, Sarge-Njie R, Alabi A, et al Seroprevalence of hepatitis B and C virus in HIV-1 and HIV-2 infected Gambians. Virology journal. 2010;7(1):230. 20843322 10.1186/1743-422X-7-230 PMC2949835

[27] Soriano V, Mocroft A, Peters L, Rockstroh J, Antunes F, Kirkby N, et al Predictors of hepatitis B virus genotype and viraemia in HIV-infected patients with chronic hepatitis B in Europe. Journal of antimicrobial chemotherapy. 2010;65(3):548–55. 10.1093/jac/dkp479 20051475

[28] Thio CL, Seaberg EC, Skolasky R Jr, Phair J, Visscher B, Muñoz A, et al HIV-1, hepatitis B virus, and risk of liver-related mortality in the Multicenter Cohort Study (MACS). The Lancet. 2002;360(9349):1921–6. 10.1016/s0140-6736(02)11913-1 12493258

[29] BenMarzouk-Hidalgo OJ, Torres-Cornejo A, Gutierrez-Valencia A, Ruiz-Valderas R, Viciana P, López-Cortés LF. Higher Activation in CD4+ T Cells But Similar Viral Control Among HIV/Hepatitis C Virus-Coinfected Patients on a Simplification Monotherapy. AIDS research and human retroviruses. 2016;32(1):6–11. 10.1089/AID.2014.0299 26414169

[30] Alter MJ. Epidemiology of viral hepatitis and HIV co-infection. Journal of hepatology. 2006;44:S6–S9. 16352363 10.1016/j.jhep.2005.11.004

